# Modeling Drug and Radiation Resistance with Patient-Derived Organoids: Recent Progress, Unmet Needs, and Future Directions for Lung Cancer

**DOI:** 10.3390/cells14241994

**Published:** 2025-12-15

**Authors:** Dahye Lee, Yoonjoo Kim, Da Hyun Kang, Chaeuk Chung

**Affiliations:** Division of Pulmonology and Critical Care Medicine, Department of Internal Medicine, College of Medicine, Chungnam National University, Daejeon 34134, Republic of Korea; ziczi02@naver.com (D.L.); misspims@naver.com (Y.K.)

**Keywords:** lung cancer, acquired resistance, chemotherapy resistance, radiotherapy resistance, patient-derived organoid, tumor microenvironment, immune checkpoint inhibitors, antibody-drug conjugates, single cell RNA sequencing, spatial transcriptomics

## Abstract

**Highlights:**

**What are the main findings?**

- Patient-derived organoids (PDOs) faithfully recapitulate the genetic heterogeneity and histological architecture of original tumors, serving as a superior platform for modeling intrinsic and acquired resistance mechanisms, particularly the reversible state of drug-tolerant persisters (DTPs).
- Despite their utility, current PDO models face significant challenges, including the absence of tumor microenvironment (TME) components, the lack of standardized drug sensitivity cutoff values, and the scarcity of acquired resistance models in lung cancer due to re-biopsy limitations.

**What is the implication of the main finding?**

- Integrating PDOs with advanced technologies—such as single-cell RNA sequencing, spatial transcriptomics, and immune co-culture systems—enables the precise mapping of resistance evolutionary trajectories and the evaluation of novel therapeutics like antibody-drug conjugates (ADCs).
- Establishing standardized culture protocols and large-scale biobanks will transition PDOs from experimental tools to robust “pharmacotyping” platforms, facilitating functional precision medicine and improved treatment strategies for relapsed lung cancer patients.

**Abstract:**

**Background:** Chemotherapy, targeted therapy and radiotherapy are the cornerstones of cancer treatment. However, therapeutic resistance—not only to these classic modalities but also to novel therapeutics like immune checkpoint inhibitors (ICIs) and antibody-drug conjugates—remains a major hurdle. Resistance significantly limits efficacy and increases recurrence rates. A deep understanding of the molecular mechanisms driving this resistance is critical for developing personalized therapeutic strategies and improving patient outcomes. **Recent Advances:** Patient-derived cancer organoids have emerged as a powerful preclinical platform that faithfully recapitulates the genetic, phenotypic, and histological characteristics of original tumors. Consequently, PDOs are being widely utilized to evaluate drug responses, investigate resistance mechanisms, and discover novel therapeutic targets for a range of therapies. **Limitations:** While organoid models have been instrumental in studying resistance, significant limitations persist. First, standard organoid-only models lack key tumor microenvironment components, such as immune cells, limiting immunotherapy research. Second, there is a significant lack of research on acquired resistance, particularly in lung cancer. This gap is largely driven by the clinical infeasibility of rebiopsy in patients with progressive diseases. Third, the absence of standardized protocols for generating and validating resistance models hinders reproducibility and complicates clinical translation. **Conclusions:** This review summarizes recent advances in using organoid models to study resistance to chemotherapy, radiotherapy, and novel therapeutics (ICIs and ADCs). We emphasize the critical need for standardization in resistance organoid research. We also propose future directions to overcome existing challenges, including the integration of co-culture systems (to include the TME) and advanced technologies (e.g., scRNA-seq, Spatial Transcriptomics). Our specific focus is on advancing lung cancer resistance modeling to enable functional precision medicine.

## 1. Introduction

Despite significant advances in cancer therapies, therapeutic resistance remains the greatest obstacle to curing cancer. Multidrug resistance (MDR), in particular, is a pervasive clinical challenge, contributing to the vast majority of cancer-related mortality [1,2,3].

Lung cancer is the leading cause of cancer-related mortality worldwide, making it a highly fatal disease. Although a substantial portion of patients with non-small cell lung cancer (NSCLC) initially respond well to chemotherapy or targeted therapy, the vast majority inevitably develop acquired resistance. This ultimately leads to disease relapse and mortality. Consequently, overcoming the mechanisms of this acquired resistance remains the most urgent and critical clinical challenge for fundamentally improving the survival rates of lung cancer patients [4,5,6,7].

**Figure 1:**
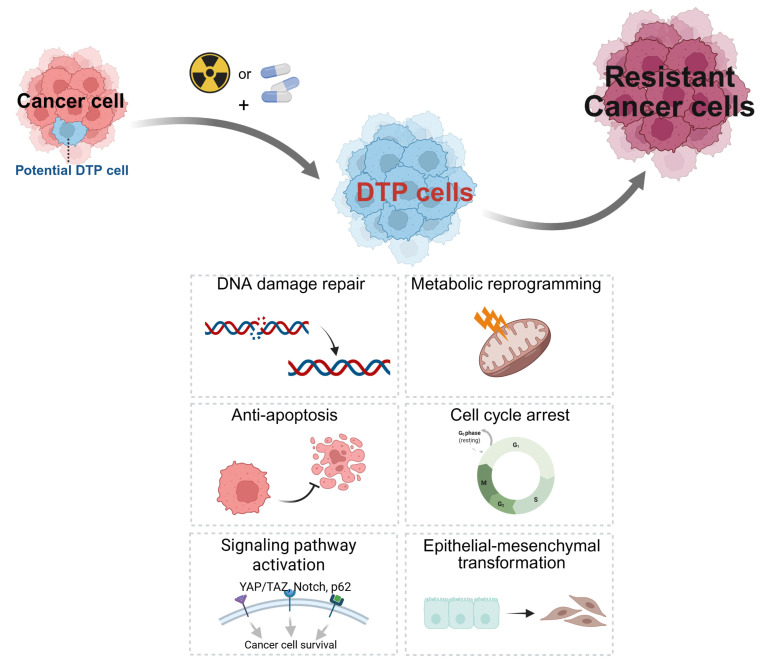
Molecular mechanisms of resistance to drugs and radiotherapy. [Original illustration by the authors] Figure 1 illustrates the process by which cancer cells acquire resistance during Drug or radiotherapy. (1) While most cancer cells are killed during treatment, some potential DTP cells transition into a DTP cell state and survive. (2) These DTP cells activate various molecular mechanisms for survival, such as DNA damage repair, metabolic reprogramming, epigenetic adaptation, anti-apoptosis, cell cycle arrest (quiescence), activation of survival signaling pathways (e.g., YAP/TAZ, AXL), and epithelial–mesenchymal transformation (EMT). It is important to note that while the DTP state is often reversible and epigenetically driven, continuous therapeutic pressure facilitates the fixation of these traits, ultimately (3) forming a population of resistant cancer cells that are non-responsive to treatment. Created in BioRender. Lee, D. (2025) https://BioRender.com/6vl2w2z.

This acquired resistance arises through a complex process by which cancer cells adapt to therapeutic pressure. During the initial treatment, while most cancer cells are eliminated, a small population of drug-tolerant persister cells survives. These DTP cells evade the initial drug pressure not through pre-existing genetic mutations, but through reversible, non-genetic mechanisms. This phenomenon strikingly parallels the development of antimicrobial resistance in pathogens, where sub-populations of bacteria (“persisters”) survive antibiotic treatment through metabolic dormancy or biofilm formation to eventually repopulate the colony [8,9]. In cancer, these adaptive mechanisms include epigenetic reprogramming (e.g., altered histone methylation/acetylation), metabolic shifts toward oxidative phosphorylation or fatty acid oxidation, and the activation of bypass signaling pathways such as AXL, FGFR, or YAP/TAZ signaling [10,11,12]. Furthermore, these cells often undergo Epithelial-to-Mesenchymal Transition (EMT), characterized by the upregulation of transcription factors like ZEB1 and TWIST, which confers a survival advantage against cytotoxic stress [10]. This DTP population then serves as a reservoir from which fully resistant clones emerge through the eventual acquisition of stable genetic mutations or permanent epigenetic changes (Figure 1).

Traditional cancer research platforms, including 2D cell culture systems, genetically engineered mouse models, and patient-derived xenograft (PDX) models, have been indispensable for drug development. However, these models have inherent limitations in accurately replicating the complex tumor microenvironment, intratumoral heterogeneity, and patient-specific immune responses [13,14].

To address these shortcomings, organoid technology has emerged as a transformative alternative. Patient-derived organoids are three-dimensional cellular structures derived from patient tissues that faithfully maintain the genetic and histological features of the original tumors [15,16,17,18,19]. This high fidelity makes them invaluable platforms for evaluating drug responses, investigating resistance mechanisms, and identifying new therapeutic targets [20,21,22,23].

To fully appreciate the utility of PDOs, it is necessary to contextualize them within the spectrum of available preclinical models. Traditional 2D cell lines are cost-effective and easy to manipulate but fail to recapitulate the spatial architecture and heterogeneity of tumors. Conversely, Patient-Derived Xenograft models preserve the tumor architecture but are limited by low take rates, long establishment times (often exceeding the clinical decision window), and the substitution of human stroma with murine components [13,14]. Organ-on-a-chip models offer high physiological relevance but are currently lower throughput and technically complex. PDOs occupy a “sweet spot” among these models: they are more physiologically relevant than 2D cultures, more scalable and faster to establish than PDX models, and amenable to high-throughput screening, although they currently lack the systemic complexity of in vivo models.

In particular, Lung Cancer Organoid (LCO) models are being actively utilized as a key platform to study complex resistance mechanisms, analyze interactions with the tumor microenvironment (TME) and tumor macroenvironment (TMaE), and realize patient-specific precision medicine [24,25].

Recently, innovative therapeutics such as immune checkpoint inhibitors and antibody-drug conjugates have been introduced in the clinic, significantly improving treatment outcomes. However, resistance to these novel agents is also emerging as a major challenge. Investigating these complex resistance mechanisms necessitates robust preclinical models that can faithfully recapitulate the characteristics of the original tumor [26,27,28,29].

**Figure 2:**
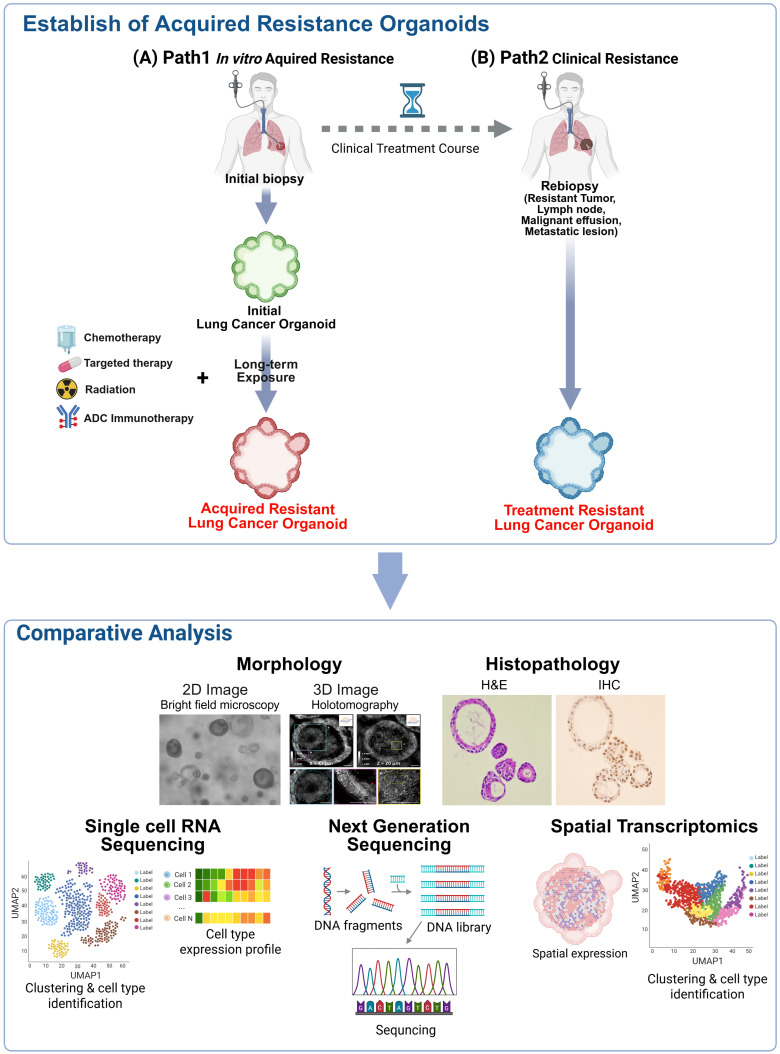
Establishment and multi-faceted comparative analysis of acquired resistance organoid models. [Original illustration by the authors] Figure 2 illustrates two primary workflows for generating resistance models and how they differentiate between intrinsic and acquired resistance. (**A**) Direct Generation from Resistant Patients: Organoids are established from biopsies taken after a patient has developed clinical resistance. This approach models intrinsic or pre-existing resistance mechanisms already present at the time of the second biopsy. (**B**) Long-term Drug Exposure in vitro: Sensitive organoids from treatment-naïve patients are subjected to prolonged, escalating doses of anticancer drugs to experimentally induce resistance. This method enables the longitudinal modeling of the acquired resistance process, allowing for the tracking of evolutionary trajectories from a sensitive state to a fully resistant phenotype. Both approaches allow for comprehensive downstream analyses, including drug screening, genomics (WES/WGS), transcriptomics (RNA-seq, scRNA-seq), and proteomics, to elucidate resistance mechanisms. The images demonstrate (**a**) mucosal layer, (**b**) apicobasal polarization, ciliated cells (red arrowed) and (**c**) various intracellular organelles, such as mitochondria and lipid droplets. Created in BioRender. Lee, D. (2025) https://BioRender.com/89gwr5n.

In resistance studies, organoids are typically established in two ways: either by culturing tumor tissues from clinically resistant patients [30,31,32,33] or by inducing resistance in sensitive organoids through prolonged exposure to anticancer drugs or radiation, as well as next-generation therapeutics such as ICIs and ADCs [34,35,36]. While these approaches have been successfully applied across various cancer types, research focusing on lung cancer, especially acquired resistance, remains notably limited. Moreover, the lack of standardized protocols and quality control criteria for generating resistant organoids creates significant challenges in reproducibility and reliability (Figure 2).

In this review, we summarize the current advances in generating and applying organoid models of resistance to chemotherapy, targeted therapy, radiotherapy, and next-generation therapeutics, including ICIs and ADCs. We further underscore the urgent need for organoid-based resistance studies in lung cancer and discuss future research directions and potential clinical applications.

## 2. Overview of Organoid Technology

Organoids are three-dimensional cellular structures, cultured from stem cells or patient-derived tissues, that self-organize to mimic the structural and functional characteristics of the source organ, effectively serving as “mini-organs.” Cancer organoids, in particular, recapitulate the genetic heterogeneity, tissue architecture, and treatment response profiles of the parental tumors with much higher fidelity than traditional 2D cultures or PDX models [14]. This makes them an ideal platform for drug screening, combination therapy experiments, and elucidating mechanisms of drug resistance, positioning them as an invaluable tool for personalized precision medicine [15,17,19,21,23,35,36].

In 2009, Sato and colleagues pioneered the long-term culture of mouse intestinal organoids by identifying the key niche factors required for intestinal stem cell growth [37]. This breakthrough was followed in 2011 by their successful development of human colon cancer organoids from patient tissues [38], paving the way for the widespread adoption of organoid models across various human organs and tumor types.

Organoids can be broadly categorized by their origin:

Adult stem cell-derived organoids are generated directly from resected tumor tissues or biopsies. They preserve the tissue-specific identity and genetic stability of the original tissue, making them widely used for modeling disease and regeneration. Notably, PDOs retain cancer-specific mutations and intratumoral heterogeneity with high success rates and scalability, making them a cornerstone of modern cancer research [18,36,39,40,41].

Induced pluripotent stem cell (iPSC)-derived organoids can recapitulate developmental processes and are extensively utilized in modeling genetic disorders [41,42,43,44].

Despite these advantages, most organoid cultures are established in isolation, lacking essential components of the TME such as immune cells, cancer-associated fibroblasts (CAFs), and vasculature [45,46]. This limits their ability to fully capture complex immune responses and stromal interactions. However, a key advantage of the 3D organoid platform is its inherent scalability to overcome this limitation. Unlike 2D cultures, organoids readily support co-culture with other TME components, such as patient-derived immune cells or CAFs, holding the potential to create more physiologically relevant tumor models. The integration of these co-culture systems with emerging technologies like organ-on-a-chip platforms is being developed to enable more physiologically relevant tumor modeling [47,48]. The integration of organoid technology with single-cell transcriptomics, CRISPR-based functional genomics, and high-throughput drug screening has further expanded its utility [49,50,51,52,53]. Furthermore, the establishment of organoid biobanks is accelerating preclinical and translational research by enabling patient-specific drug response prediction and biomarker discovery [19,54,55,56].

## 3. Recent Advances in Organoid-Based Studies of Drug and Radiotherapy Resistance

Despite advances in therapeutic strategies, many patients eventually develop resistance, leading to reduced efficacy, shortened progression-free survival, and disease relapse. Organoids provide a unique platform to model both intrinsic and acquired resistance, enabling active investigation into their underlying mechanisms.

### 3.1. Organoids for Modeling Drug Resistance

Chemotherapy and targeted therapy resistance are primary obstacles in cancer treatment. In contrast to traditional models, PDOs preserve tumor heterogeneity and patient-specific genetic traits, making them superior platforms for studying both intrinsic and acquired resistance mechanisms (Table 1).

Numerous studies have leveraged organoids to explore chemotherapy resistance:

In ovarian cancer, a comparison of PDOs from Cisplatin-sensitive and -resistant patients revealed that resistant organoids overexpressed Aurora A. This kinase activated the SOX8-FOXK1 signaling pathway, which in turn regulated genes involved in cell senescence and glycolysis, identifying the Aurora A–SOX8–FOXK1 axis as a key driver of cisplatin resistance [57]. Another study showed that patient-derived ovarian cancer organoids faithfully recapitulate the genetic features of primary tumors, providing a valuable model system for evaluating drug sensitivity and resistance. Specifically, it demonstrates differences in sensitivity and resistance to platinum-based chemotherapy and PARP inhibitors, highlighting their potential for personalized treatment strategy development [58].

**Table 1:** Chemotherapy resistance organoid models.

Cancer	Drug Used	Organoid Type	Key Finding	Reference
Colorectal cancer	Oxaliplatin	Intrinsic/Acquired	Lnc-RP11-536K7.3/SOX2–HIF1α pathway drives Oxaliplatin resistance; knockout restores sensitivity.	[34]
	5-FU, Oxaliplatin, Irinotecan, Paclitaxel	Acquired	PDO drug response strongly correlated with clinical outcomes (100% sensitivity for paclitaxel).	[59]
	Irinotecan (monotherapy), 5-FU + Irinotecan (FI)	Intrinsic	PDO-based test successfully predicted clinical response (non-responders) to irinotecan-based therapies.	[60]
Gastric cancer	5-FU	Acquired	KHDRBS3 upregulation induced 5-FU multidrug resistance and cancer stem cell features.	[61]
	Oxaliplatin	Acquired	Myoferlin (MYOF) upregulation drove acquired oxaliplatin resistance; knockdown restored sensitivity.	[62]
	37 anticancer drugs (HTS)	Intrinsic (Biobank)	Established a large biobank capturing GC molecular subtypes. HTS identified novel drug sensitivities and linked ARID1A mutations to increased sensitivity to ATR inhibitors (VE-822).	[54]
Ovarian cancer	Cisplatin	Intrinsic/Acquired	Aurora A–SOX8–FOXK1 axis promoted Cisplatin resistance via senescence/glycolysis regulation.	[57]
	Cisplatin, others (X22)	Intrinsic/Acquired	PDOs recapitulated tumor genetics; useful for evaluating sensitivity to platinum and PARP inhibitors.	[58]
Pancreatic cancer	Gemcitabine, others (X 5)	Intrinsic	Pharmacotyping of 66 PDOs correlated with clinical response; tracked resistance evolution.	[30]
	Gemcitabine, Paclitaxel	Intrinsic	Identified BARD1, RAD50, and other genes as potential biomarkers for Gem/Paclitaxel resistance.	[31]
	Gemcitabine, 5-Fluorouracil, Paclitaxel	Intrinsic (PDO-CAF Co-culture)	CAF co-culture induced chemoresistance and an EMT phenotype in PDOs.	[63]
Esophageal cancer	Cisplatin + 5-FU (CF)	Intrinsic/Acquired	Resistant PDOs showed NRF2 pathway hyperactivation; Fedratinib identified as potential therapy.	[64]
	Cisplatin, others (X4)	Intrinsic	PDO drug sensitivity profiles correlated with patient clinical outcomes.	[32]
Head and neck squamous cell carcinoma	Nutlin-3a, Alpelisib, PRMT5 inhibitor (EZP015566), Radiotherapy (RT)	Intrinsic (Biobank)/Gene-edited	PDO response to RT correlated with patient relapse. HTS linked CDKN2A loss to PRMT5 inhibitor sensitivity.	[51]
Lung cancer	EGFR-TKI, Cisplatin	Intrinsic	PDO drug response correlated with patient clinical outcomes for targeted therapies (e.g., Src activation).	[65]
	Osimertinib, others (X7)	Acquired	PDOs effectively predicted chemotherapy and targeted therapy resistance, reflecting clinical responses.	[33]

5-FU, 5-Fluorouracil; PDOs, Patient derived organoids; TKIs, Tyrosine Kinase Inhibitor; HTS, High-throughput screening; CAFs, cancer-associated fibroblasts; EMT, Epithelial-to-mesenchymal transition.

In colorectal cancer, the long noncoding RNA, Lnc-RP11-536K7.3, was upregulated in oxaliplatin-resistant PDOs. This lncRNA promoted chemoresistance via the SOX2–HIF-1α signaling pathway, and its knockout restored Oxaliplatin sensitivity, demonstrating a clear therapeutic target [34]. In a separate study by Vlachogiannis et al., PDOs from metastatic colorectal and gastroesophageal cancer patients showed drug responses that strongly correlated with clinical outcomes. Notably, the model predicted paclitaxel response with 100% sensitivity and 93% specificity, highlighting its value for patient-tailored treatment selection. This high correlation was also observed between the PDO responses and in vivo results from corresponding patient-derived organoid xenograft (PDOX) models [59]. Similarly, Ooft et al. demonstrated that PDOs from metastatic CRC patients could predict clinical responses to irinotecan-based therapies [60].

In gastric cancer, 5-FU resistance was induced in PDOs, leading to elevated expression of KHDRBS3, which promoted multidrug resistance and stem cell-like features [61]. Similarly, Oxaliplatin-resistant organoids showed significant upregulation of Myoferlin (MYOF). MYOF inhibition restored drug sensitivity, and its high expression correlated with poor patient prognosis, suggesting its potential as both a biomarker and a therapeutic target [62]. Further studies have used gastric cancer PDOs to link specific genotypes to drug sensitivity, such as identifying ARID1A mutations as a predictive biomarker for sensitivity to the ATR inhibitor VE-822 [54].

In pancreatic cancer, pharmacotyping of 66 PDOs revealed a strong correlation between their drug response profiles and patient clinical outcomes. This study used longitudinal PDO analysis to track the evolution of acquired resistance, identifying chemosensitivity gene signatures that could predict treatment responses [30]. Omaya et al. demonstrated that PDOs can be used to predict chemotherapy resistance, identifying genes like BARD1, RAD50, SLC25A10, and MAP3K9 as potential biomarkers for resistance to gemcitabine and gemcitabine + paclitaxel, making PDOs a valuable model for evaluating drug sensitivity in pancreatic cancer [31]. Furthermore, studies integrating the TME have shown that co-culture with cancer-associated fibroblasts (CAFs) can induce chemoresistance via mechanisms like exosome secretion [63].

In esophageal squamous cell carcinoma, resistant organoids exhibited hyperactivation of the NRF2 signaling pathway. High-throughput screening (HTS) on this platform identified Fedratinib as a promising therapeutic candidate for resistant cases. Furthermore, this study demonstrated that their PDOX models successfully recapitulated the unique histopathological and genetic characteristics of the original tumors [64]. Another study showed that PDOs can be used to assess chemotherapy resistance, with drug sensitivity profiles correlating with clinical outcomes, highlighting their potential to guide personalized treatment strategies for patients [32].

In head and neck squamous cell carcinoma, PDOs have been used to link TP53 mutation status to Nutlin-3a sensitivity and CDKN2A loss to sensitivity against PRMT5 inhibitors, validating their use for biomarker discovery [51].

While these findings in colorectal and pancreatic cancers are promising, they highlight a specific gap in lung cancer research. The protocols successfully applied to track resistance evolution in gastrointestinal organoids serve as a critical roadmap for lung cancer, where acquired resistance models to platinum-based chemotherapy—a standard-of-care for a large proportion of patients—remain notably absent.

In lung adenocarcinoma, Kim et al. demonstrated a strong correlation between PDO drug responses and patient clinical outcomes for targeted therapies. This study confirmed the utility of PDOs for predicting treatment efficacy and began to elucidate mechanisms of acquired resistance, such as Src activation [65]. However, this work focused primarily on tyrosine kinase inhibitors (TKIs). Models of acquired resistance to platinum-based chemotherapy—a standard-of-care for a large proportion of lung cancer patients—remain notably absent. This represents a critical gap in our ability to address a major cause of treatment failure in lung cancer. Wang et al. discovered that PDOs can effectively predict chemotherapy and targeted therapy resistance, with drug sensitivity tests accurately reflecting clinical responses and aiding in the development of personalized treatment strategies for various subtypes of advanced lung cancer [33].

Collectively, these studies unequivocally demonstrate that organoids are a powerful platform for elucidating complex resistance pathways, identifying prognostic biomarkers, and evaluating novel therapeutic strategies to overcome resistance.

### 3.2. Organoids for Modeling Radiotherapy Resistance

Radiotherapy is a critical treatment modality for many solid tumors, but intrinsic or acquired radioresistance is a primary cause of local recurrence and treatment failure [66]. PDOs are now being utilized to predict patient-specific radiosensitivity and investigate the mechanisms of radioresistance.

In rectal cancer, PDO radiosensitivity demonstrated high concordance with patients’ actual clinical outcomes. Transcriptomic profiling revealed that the activation of DNA damage repair pathways, particularly the ATM/ATR signaling cascade, was a core mechanism of radioresistance in these models. This concordance in chemoradiation (CRT) response was likewise confirmed in PDOX models [67]. Another study discovered that rectal cancer organoids can effectively model radiotherapy resistance, showing that radiation-resistant organoids have enhanced DNA repair pathways and antioxidant metabolism. Treatment with GCLC inhibitors and RRx-001 induced oxidative stress and improved radiotherapy sensitivity, overcoming resistance [68]. Andel et al. showed that rectal cancer organoids can model radiotherapy resistance, with radiation-resistant organoids showing enhanced subclone survival and reduced chromosomal instability, indicating that subclonal populations are crucial in the development of radioresistance [69].

In colorectal cancer, organoid models were used to show that thymoquinone (TQ) acts as a radiosensitizer. The combination of TQ and radiation suppressed key radioresistance pathways, including NF-κB and Wnt/β-catenin signaling, and decreased the expression of cancer stem cell markers, thereby inhibiting tumor regenerative capacity [70].

In glioma, a PDO model spatially pinpointed cancer stem cell (CSC) niches as hubs of radioresistance, which were characterized by enhanced DNA damage repair and hypoxia-induced survival signaling. These findings were recapitulated in the corresponding PDOX models. his provided a novel platform to mechanistically investigate and target these resilient subpopulations [71].

In nasopharyngeal carcinoma, PDOs generated under hypoxic conditions modeled intrinsic radioresistance by upregulating HIF-1α. This study demonstrated the potential of PDOs as a preclinical platform for optimizing radiation dosage and developing personalized radiotherapy strategies [72]. Issing et al. demonstrated that head and neck cancer organoids can effectively model radiotherapy resistance, showing that HPV infection and TP53 mutations significantly influence the radiation response, with HPV-positive tumors exhibiting greater sensitivity to radiation, while TP53 mutations correlate with increased radioresistance [73].

Pancreatic cancer organoids can also model radiotherapy resistance, with varying sensitivity to radiation observed in the dose–response relationship across different organoids. One PDO showed higher radiation sensitivity, which can be used to predict individual radiation therapy responses, suggesting that PDOs can be effectively utilized in personalized treatment planning [74].

In lung cancer, the dose-treatment response relationship of radiation was evaluated using patient-derived tumoroids, and DNA damage and cell death were analyzed through cell viability assays and immunofluorescence staining. Sensitivity to treatment varied depending on the radiation dose, and it was found that radiation-resistant organoids exhibited less DNA damage and enhanced survival [75].

Thus, organoids are gaining recognition as a valuable platform for understanding the biological mechanisms of radioresistance and for discovering novel radiosensitizing strategies (Table 2). However, challenges such as the lack of standardization in irradiation methods, fractionation schedules, and long-term follow-up protocols remain key issues that must be addressed in future research.

### 3.3. Modeling Resistance to Novel Therapeutics: ICIs and ADCs

In recent years, ICIs have revolutionized the paradigm of lung cancer treatment. However, many patients either do not respond from the outset (intrinsic resistance) or develop resistance after an initial response (acquired resistance) [26,27,76]. Response and resistance to immunotherapy are heavily dependent not only on the intrinsic characteristics of the tumor cells but also on complex interactions with immune cells within the TME. Consequently, traditional organoid models, which lack immune components, have had significant limitations for immunotherapy research. However, this limitation is being overcome by advances in co-culture techniques that enable the cultivation of tumor cells together with autologous immune cells derived from the patient’s tumor tissue [77,78].

Neal et al. established a co-culture system of patient-derived tumor organoids with autologous immune cells, enabling the modeling of tumor–immune interactions. Critically, this model allowed for the mechanistic dissection of PD-1 blockade efficacy, demonstrating that successful treatment led to the reinvigoration of exhausted CD8+ T cells (Tex), characterized by enhanced cytotoxicity and the increased secretion of effector cytokines such as IFN-γ and TNF-α. Conversely, resistant models exhibited persistent T-cell dysfunction and upregulation of alternative checkpoint ligands [79]. Building on these advances, Li et al. recently introduced a gel–liquid interface (GLI) co-culture system combining lung cancer organoids with autologous peripheral blood mononuclear cells (PBMCs), which recapitulated patient-specific responses to PD-1 blockade and profiled systemic tumor-reactive effector memory-like T cells [46]. However, the application of such complex co-culture models to systematically investigate acquired ICI resistance in lung cancer remains highly limited, representing a significant research gap.

Furthermore, antibody-drug conjugates, which are emerging as a next-generation therapeutic, deliver potent cytotoxic payloads to tumor cells by binding to specific target antigens [80]. In the context of lung cancer, ADCs such as Trastuzumab deruxtecan and Patritumab deruxtecan are being actively used in the clinic [81,82]. Although the application of ADCs in organoid systems remains in its infancy, a small number of studies have demonstrated the feasibility of evaluating Sacituzumab govitecan responsiveness using patient-derived organoids in metastatic breast cancer and cholangiocarcinoma. These proof-of-concept studies highlight the potential utility of organoids not only for profiling the efficacy and toxicity of ADCs, including interstitial lung disease (ILD), but also for investigating emerging adaptive responses within a physiologically relevant three-dimensional context [83,84]. However, studies specifically investigating ADCs’ resistance mechanisms utilizing organoid models across diverse cancer types remain limited. Considering the trend of rapid clinical adoption of ADCs, organoid-based platforms are highlighted for their value as crucial and high-potential preclinical models to accelerate ADC development and address potential resistance issues.

## 4. Clinical Trials Involving Cancer Organoids Resistant to Drug or Radiotherapy

Recent studies have highlighted the central role of patient-derived organoids in drug sensitivity and resistance research, particularly in predicting treatment responses and designing personalized therapies for metastatic or relapsed-resistant cancers. As a result, clinical translation studies using PDOs have become increasingly active. PDOs are especially recognized as a practical platform for studying treatment resistance in high-grade cancers that exhibit resistance to chemotherapy or radiation therapy, such as high-grade serous ovarian cancer, lung cancer, head and neck cancer, and glioblastoma (Table 3).

Specifically, clinical trials using PDOs focus on two main approaches. One involves drug screening within PDOs to identify chemotherapy or radiation sensitivity profiles, which can suggest more effective treatment combinations for individual patients. The other approach experimentally validates the mechanisms of resistance acquired by tumor cells after recurrence or multi-line treatments (e.g., glioma stem cells, hypoxic microenvironment, changes in microvascular structure) using PDOs. This trend demonstrates that research has progressed beyond simple sensitivity testing and is now entering the stage of designing ‘resistance-overcoming therapeutic strategies’.

## 5. Key Challenges and Unmet Needs in Lung Cancer Resistance Research

While the utility of organoids in modeling resistance has been demonstrated across various cancer types, significant hurdles remain. These challenges are particularly pronounced in the establishment of high-purity resistant organoids, and include optimizing culture media, maintaining tumor heterogeneity, and shortening the long timeline required for their generation. Moreover, gradual genomic and phenotypic drift during serial passaging may alter drug response trajectories, underscoring the need for routine genomic benchmarking against matched primary tumors. However, despite their promise, PDOs are not without significant limitations that must be addressed for broad clinical application.

### 5.1. Technical Limitations and Standardization Issues

Although organoids are widely used in cancer research, reproducibility between laboratories is often limited by variations in media compositions and culture conditions [85,86]. A critical technical limitation is the reliance on animal-derived basement membrane extracts (e.g., Matrigel) as a scaffold. These matrices suffer from batch-to-batch variability in composition and stiffness, which can significantly alter drug sensitivity and cellular phenotype, complicating the interpretation of resistance mechanisms.

Furthermore, standard organoid cultures primarily consist of epithelial tumor cells and lack the complex tumor microenvironment. The absence of immune cells, cancer-associated fibroblasts, and vasculature means that standard PDOs cannot model extrinsic resistance mechanisms—such as immune evasion or stroma-mediated drug protection—without the introduction of complex co-culture systems [45,46,63].

Additionally, a significant limitation lies in the spatial and temporal differences between organoids and real tumors regarding therapeutic delivery. In patients, drug delivery is influenced by vascular density, blood flow, and high interstitial fluid pressure. In contrast, organoids rely on passive diffusion from the culture medium, which may create artificial concentration gradients that do not fully reflect the physiological barriers to drug penetration found in vivo [87,88,89]. Similarly, while organoids can model cellular radiosensitivity, they cannot capture the complex dosimetry and tissue penetration depth issues inherent to clinical radiotherapy, nor can they fully model the radio-protective effects of surrounding normal tissues.

#### Challenges Specific to Lung Cancer

This problem is especially acute for lung cancer organoids, which face unique challenges. A primary difficulty is obtaining sufficient tissue for culture initiation. Furthermore, the rapid overgrowth of normal bronchial epithelial cells frequently contaminates cultures, drastically reducing the success rate of establishing pure cancer organoids. Consequently, reported establishment efficiencies vary widely, from as low as 7% to 92% [90]. This variability highlights a major translational barrier: if a model cannot be reliably established for the majority of patients, its utility as a universal diagnostic tool is limited.

Another major issue is the morphological similarity between normal bronchial and cancerous organoids, which makes simple microscopic observation insufficient for distinguishing them. This necessitates additional validation steps, such as immunohistochemistry staining for tumor-specific markers. To address the contamination issue, recent protocols propose modified media formulations that selectively inhibit normal cell proliferation, for instance, by withdrawing key growth factors like Wnt3a, Noggin, and A83-01 [91,92,93,94]. Interestingly, samples derived from malignant pleural effusions or metastatic sites often yield higher-purity cancer organoids, likely due to the lower probability of contamination with normal tissue [33,95].

### 5.2. Clinical Translation Barriers

Beyond technical issues, real-world constraints hinder the routine clinical use of PDOs. The most critical factor is the “turnaround time.” Establishing a sufficient number of organoids for drug screening typically takes 4–8 weeks [23,90]. For patients with advanced NSCLC, who often require immediate second-line treatment upon progression, this timeline may be too slow to inform clinical decision-making [56]. Additionally, the high cost of growth factors and sequencing analysis poses an economic barrier to widespread adoption in public healthcare systems [85,86].

Furthermore, rigorous validation of the model’s predictive accuracy is a prerequisite for clinical adoption. While many studies report high concordance between organoid responses and patient outcomes, standardized criteria for defining “resistance” in vitro are still lacking. Unlike bacterial antibiotic susceptibility testing (e.g., MIC values), there are no universally established IC50 cutoff values to distinguish sensitive from resistant organoids. Establishing these thresholds and validating the positive predictive value and negative predictive value through large-scale prospective clinical trials are urgent unmet needs.

In resistance studies, a common approach is to analyze organoids derived directly from resistant patients or to use short-term drug exposure to study acute responses [34,57,62]. However, this fails to capture the dynamic, multi-step process of acquired resistance that evolves gradually over the course of treatment. The lack of standardized protocols for systematically generating and validating long-term acquired resistance organoids results in low consistency across studies and severely limits their clinical translation.

Additionally, extracellular matrix composition and biomechanical stiffness can significantly influence drug tolerance, yet current Matrigel-based platforms remain poorly defined and difficult to standardize across laboratories [16,96].

### 5.3. Critical Research Gaps in Lung Cancer Resistance

Despite established standard-of-care treatments for NSCLC, resistance is a near-inevitable event that leads to recurrence and metastasis [4,7]. Unlike other cancer types such as colorectal and ovarian cancer, where research using acquired resistance organoids is active, there is a striking lack of such models for lung cancer. Although clinical resistance to targeted therapy, chemotherapy, immunotherapy, and radiotherapy is frequently observed, very few studies have systematically modeled the temporal dynamics and molecular pathways of resistance acquisition at the organoid level.

A major clinical barrier to studying acquired resistance is the limitation of rebiopsy. Recent advancements in diagnostic and interventional pulmonology, such as robotic bronchoscopy and the development of thin and ultra-thin cryobiopsy probes, have significantly enhanced the success rate of rebiopsy procedures [97,98]. However, in cases where a patient’s overall condition deteriorates due to prior treatments or disease progression, or when tumors are located in inaccessible sites such as the brain or bones, rebiopsy may not be feasible [93,95].

Therefore, organoid-based resistance modeling becomes essential to investigate resistance mechanisms and test alternative therapeutic regimens without additional invasive procedures. Without such models, oncologists are often forced to rely on empirical treatment sequencing with limited evidence, highlighting the critical and immediate importance of a platform that can recapitulate patient-specific resistance pathways in vitro.

To overcome rebiopsy limitations, recent studies have explored generating organoids from malignant effusions or circulating tumor cells, offering a minimally invasive approach to capture evolving resistance phenotypes longitudinally. However, such models may not fully recapitulate the spatial heterogeneity and microenvironmental influences of the primary tumor, and the cellular composition of effusion-derived samples can shift toward more aggressive or mesenchymal states, introducing potential biases in resistance interpretation.

The biological characteristics of the rare cell populations that survive initial treatment—often termed drug-tolerant persisters—remain poorly understood. Recent reviews, such as that by Izumi et al., highlight that these DTP cells evade initial drug pressure through mechanisms including slow cell cycling, apoptosis evasion, and metabolic reprogramming, serving as a reservoir from which fully resistant clones eventually emerge [85]. The specific features and vulnerabilities of DTPs are under active investigation, as targeting this transient state before irreversible resistance develops is considered a critical therapeutic strategy. We know little about the specific mechanisms that govern the survival and subsequent proliferation of these cells under continuous therapeutic pressure. Since the onset and molecular drivers of resistance vary significantly from patient to patient, there is a critical need for a model that allows for the longitudinal observation of resistance acquisition in sensitive organoids from the same patient.

## 6. Overcoming Technical Limitations and Future Directions

### 6.1. Integrating Advanced Technologies to Overcome Limitations

To model the acquisition of resistance more accurately, it is essential to establish protocols for the long-term, sequential treatment of sensitive lung cancer organoids with anticancer drugs or radiation. This would enable a comparative analysis of organoids before and after resistance develops. By integrating this approach with advanced technologies like single-cell RNA sequencing (scRNA-seq) and spatial transcriptomics (ST), researchers can dissect the gene expression patterns of individual cells, identify specific cellular subtypes that drive resistance, and illuminate key biological mechanisms such as the activation of cancer stem cells.

For instance, a concrete workflow would involve performing scRNA-seq at three distinct timepoints: (1) the treatment-naïve state, (2) the residual disease state (DTP phase), and (3) the fully resistant state. By applying trajectory inference analysis to this data, researchers can map the evolutionary path of resistant clones, identifying whether resistance emerges from the selection of pre-existing rare subclones or through transcriptional reprogramming of the bulk population [99,100]. Furthermore, integrating spatial transcriptomics (ST) allows researchers to map these resistant subclones back to their histological context, enabling the identification of the specific spatial niches where resistant clones reside [101].

Furthermore, real tumors exist within a complex tumor microenvironment composed of fibroblasts, immune cells, and vasculature. To overcome the limitations of organoid-only models, the integration of co-culture systems or microfluidics-based organ-on-a-chip platforms is essential. In particular, models co-culturing patient-derived organoids with the patient’s own immune—such as peripheral blood mononuclear cells (PBMCs) or tumor-infiltrating lymphocytes (TILs)—show strong potential for predicting patient-specific responses to immunotherapy. These co-culture models allow for the study of complex immune resistance mechanisms, such as T-cell exhaustion, and the preemptive screening of responses to PD-1/PD-L1 inhibitors. Additionally, incorporating immunosuppressive myeloid populations such as myeloid-derived suppressor cells (MDSCs) and tumor-associated macrophages (TAMs) may further improve the ability of these platforms to model immune-mediated resistance.

### 6.2. Ultimate Goals and Vision for Research

The paramount importance of resistance organoid models lies in their potential to directly address the clinical gaps outlined above, shifting lung cancer care from a reactive to a proactive paradigm. The application of resistance organoid models has the potential to fundamentally reshape lung cancer research and treatment.

First, by using these models as a “pharmacotyping” platform to test the sensitivity of patient-derived organoids to various anticancer drugs, we can establish a robust foundation for functional precision medicine, guiding the selection of optimal treatment strategies for individual patients. This screening platform is particularly crucial for evaluating next-generation therapeutics like ADCs. Beyond assessing ADC efficacy on lung cancer organoids, this platform allows for comparison with matched patient-derived normal organoids. This enables the prediction of off-tumor toxicity and side effects such as ILD, as well as the evaluation of specificity. This provides a reliable preclinical screening platform to simultaneously validate the safety and efficacy of drug candidates during development.

Furthermore, PDOs established from initial diagnostic biopsy tissues can serve as a powerful platform to predict potential drug resistance before clinical treatment even begins. This preemptive assessment can help identify non-responders or patients at high risk of rapid resistance, aiding in the optimization of combination strategies from the start of therapy.

Another key advantage of the organoid model is the ability to conduct ‘parallel drug testing’ on the same genetic background. In the clinical setting, only one regimen can be administered to a patient at a time. However, organoid models allow for the simultaneous exposure of matched patient-derived organoids to multiple drugs, enabling direct comparison of initial response trajectories and future resistance evolution. This is particularly valuable for head-to-head testing of 3rd-generation EGFR-TKIs (e.g., Osimertinib vs. Lazertinib) or ALK inhibitors (e.g., Alectinib, Brigatinib, Lorlatinib) to determine optimal treatment sequencing and anticipate divergent resistance pathways. To translate these platforms clinically, reducing the turnaround time from biopsy to organoid expansion will be critical for ensuring that results align with real-time treatment decision windows.

**Figure 3:**
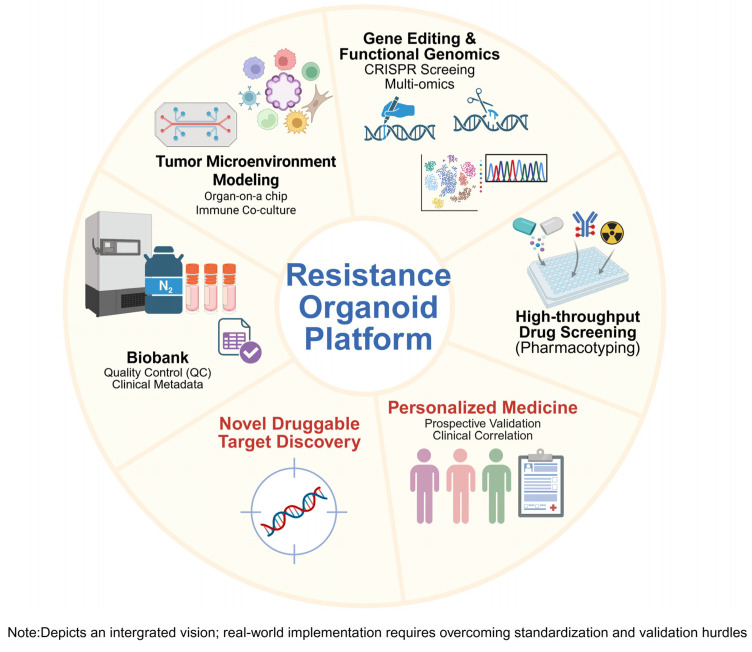
Clinical applications and future vision based on resistance organoid models. [Original illustration by the authors] The resistance organoid platform serves as a key enabling technology to realize ‘functional precision medicine’. While currently an idealized workflow, successful implementation requires overcoming challenges in standardization and turnaround time. Applications include: (1) mechanistic studies via Gene Editing, (2) High-throughput Drug Screening (Pharmacotyping), (3) Personalized Medicine (requiring prospective clinical validation), (4) Novel Druggable Target discovery, (5) Patient Biobank construction (necessitating rigorous quality control and metadata annotation), and (6) Tumor Microenvironment (TME) modeling, including the use of ‘Organ-on-a-chip’ platforms. Created in BioRender. Lee, D. (2025) https://BioRender.com/6cjr01n.

Finally, the acquired resistance organoid model serves as an invaluable discovery tool to identify novel genes, proteins, or molecular pathways that drive resistance. This, in turn, can lead to the identification of new therapeutic targets and the development of drugs designed to overcome resistance. Ultimately, this research will contribute to increasing the accuracy of treatment response prediction and improving patient survival rates through personalized therapeutic strategies. Establishing centralized, well-annotated organoid biobanks will further support multi-center validation and accelerate the translation of resistance findings into clinical practice (Figure 3).

## 7. Conclusions

Therapeutic resistance remains the primary cause of mortality in lung cancer, necessitating preclinical models that can faithfully recapitulate the complexity of tumor evolution [3,4,7]. As reviewed here, PDOs have emerged as a powerful “functional precision medicine” platform, bridging the gap between traditional 2D cultures and in vivo models [15,16,17,18,19]. By preserving the genetic heterogeneity and histological architecture of the original tumor, PDOs allow for the high-throughput screening of anticancer drugs and the mechanistic investigation of resistance pathways, particularly the transient and reversible state of DTPs [8,10].

However, significant hurdles must be overcome to translate these findings into routine clinical practice. First, standard organoid models currently lack the essential components of the TME, such as immune cells and vasculature, which limits their ability to model extrinsic resistance mechanisms and recapitulate the spatial dynamics of drug delivery found in vivo [45,46,87]. Second, technical challenges, including the variability of matrix scaffolds and the lack of standardized cutoff values for defining drug resistance, hinder reproducibility and rigorous clinical validation [16,93,102]. Third, specifically for lung cancer, there is a critical scarcity of acquired resistance models derived from re-biopsies, underscoring the need for improved establishment protocols from minimally invasive samples [90,92].

Moving forward, the next generation of organoid research must focus on integrating advanced technologies to address these limitations. The incorporation of co-culture systems and organ-on-a-chip platforms will enable the modeling of complex tumor-immune interactions and stromal influences [47,48]. Furthermore, combining longitudinal organoid sampling with scRNA-seq and spatial transcriptomics will provide unprecedented resolution in tracking the evolutionary trajectories of resistant clones [99,100,101]. Ultimately, the establishment of large-scale, well-annotated biobanks and the rigorous validation of predictive accuracy through prospective trials will be essential to realize the full potential of PDOs [54,55,56]. By shifting the treatment paradigm from reactive to proactive, organoid-based strategies hold the promise of overcoming therapeutic resistance and significantly improving survival outcomes for lung cancer patients.

## Figures and Tables

**Table 2:** Radiotherapy resistance organoid models.

Cancer	Radiation Exposure	Key Findings	Reference
Rectal cancer	Chemoradiation (CRT)	PDO radiosensitivity strongly correlated with patient clinical outcomes. Radioresistant PDOs showed activation of DNA damage repair pathways (ATM/ATR).	[67]
	Irradiation	PDOs showed enhanced DNA repair pathways and antioxidant metabolism, and treatment with GCLC inhibitor and RRx-001 induced oxidative stress and improved radiotherapy sensitivity, overcoming resistance.	[68]
	Irradiation	In rectal cancer radiotherapy-resistant organoids, enhanced survival of subclones and reduced chromosomal instability suggest that subclonal populations play a crucial role in the development of radiotherapy resistance.	[69]
Colorectal cancer	Irradiation + Thymoquinone	Thymoquinone acted as a radiosensitizer, suppressing NF-κB and Wnt/β-catenin pathways and inhibiting cancer stem cell features.	[70]
Glioblastoma cancer	Irradiation	Modeled radioresistant cancer stem cell (CSC) niches, revealing enhanced DNA damage repair and hypoxia-induced survival signaling as key resistance mechanisms.	[71]
Nasopharyngeal carcinoma (NPC)	Irradiation	Hypoxia-induced HIF-1α expression increased radioresistance. The model served as a platform for personalized radiation dose optimization.	[72]
	Irradiation	PDOs can effectively model radiotherapy resistance, and HPV infection and TP53 mutations significantly influence the radiation response.	[73]
Pancreatic cancer	Irradiation	PDOs showed varying sensitivity to radiation in the dose–response relationship, with one PDO demonstrating higher radiation sensitivity, making it useful for predicting individual responses to radiation therapy.	[74]
Lung cancer	Irradiation + Cytotoxic chemo (Cisplatin + Vinorelbine)	Patient-derived tumoroids exhibited varying sensitivity depending on the radiation dose, with radiation-resistant tumoroids showing reduced DNA damage and enhanced survival, making them a useful model for studying responses to radiotherapy.	[75]

PDOs, Patient-derived organoids.

**Table 3:** Ongoing clinical trials involving resistant cancer organoids.

Cancer	Treatment	Research Overview	Trial ID
Lung cancer	Chemotherapy	Establishment of NSCLC patient-derived organoids for drug sensitivity testing and prediction of treatment response and personalized therapy.	NCT06406608, NCT05669586
Cervical cancer	Chemotherapy + Radiotherapy	Evaluation of organoid response to chemo-radiotherapy, exploration of treatment resistance mechanisms, and identification of new therapeutic targets.	NCT06786780
Ovary cancer	Chemotherapy (Paclitaxel, Platinum–Taxane)	Development of personalized drug regimens and precision medicine approaches using ovarian cancer organoids.	NCT05813509, NCT04846933
Breast cancer	Chemotherapy	Establishment of organoid-based drug sensitivity testing and investigation of drug resistance mechanisms.	NCT03925233
Prostate cancer	Chemotherapy	Organoid-based drug sensitivity screening for castration-resistant prostate cancer with bone metastasis.	NCT06529549
Head and neck cancer	Chemotherapy	Characterization of p53-mutant tumor organoids to understand recurrence mechanisms and identify therapeutic strategies.	NCT0671941
Glioma	Chemotherapy (Temozolomide) + Radiotherapy	Use of high-grade glioma organoids for optimal drug combination screening and ctDNA/proteomics-based biomarker identification.	NCT05532397, NCT04868396

NSCLC, Non-Small Cell Lung Cancer; ctDNA, Circulating Tumor DNA.

## Data Availability

No new data were created for this review.

## Abbreviations

5-FU	5-Fluorouracil
ADC	Antibody-Drug Conjugate
CAF	cancer-associated fibroblast
CRISPR	Clustered Regularly Interspaced Short Palindromic Repeats
CRT	Chemoradiation
CSC	Cancer Stem Cell
ctDNA	Circulating Tumor DNA
DTP	Drug-Tolerant Persister
EMT	Epithelial-to-mesenchymal transition
GLI	Gel–Liquid Interface
HTS	High-throughput screening
ICI	Immune Checkpoint Inhibitor
IDLD	Interstitial Lung Disease
iPSC	Induced Pluripotent Stem Cell
lncRNA	long non-coding RNA
MDR	Multidrug Resistance
MDSC	Myeloid-Derived Suppressor Cell
NSCLC	Non-Small Cell Lung Cancer
PBMC	Peripheral Blood Mononuclear Cell
PDO	Patient-Derived Organoid
PDOX	Patient-derived organoid xenograft
PDX	Patient-Derived Xenograft
scRNA-seq	single-cell RNA sequencing
ST	Spatial Transcriptomics
TAM	Tumor-Associated Macrophage
TIL	Tumor-Infiltrating Lymphocyte
TKI	Tyrosine Kinase Inhibitor
TME	Tumor Microenvironment
